# Oct. 23. Hydrophobia

**Published:** 1849-03

**Authors:** 


*Oct.* 23. *Hydrophobia.*—Dr. Coale read the following case occurring recently in his practice.

John Fleming, a hearty full-fed boy, seven years of age, born in England but living in this country for the last four years, was brought to me one day, by his mother, with a slight laceration on the knuckle of the little finger of the left hand. The story was, that an hour before, on his way to school, he threw a stone at a dog under a wagon, and it flew out and bit him, and returned to lie down again. I saw no reason to do more than give some directions about dressing the hand very simply, and was told a few days after that it was entirely healed. Three weeks after, on Saturday Sept. 30th, his mother sent him to me about 2 P. M., saying he had been out of sorts for two or three days; and had refused to drink water for twenty-four hours. I at once determined not to favor any play to the imagination which might make this a case of hydrophobia. Looking at the throat I found it swollen somewhat, about the fauces. I asked him to take a drink on pretence of washing out his throat; but he refused it crying. An active cathartic was prescribed, with directions to let me know if he was not better in the morning. I received a message next morning to call, and found him on a little settee in the corner of the room. His look was stupid, skin cool, pulse 80. Tongue as yesterday, a little furred. The powder had acted slightly. His rest had been very slight, and much disturbed. He complained of a little pain at the epigastrium; but nowhere else, though his mother said he had complained of headache also at times. I asked him to wash his mouth out so that I could see it. He took a tablespoonful of water into his hand, and as if violently resisting and yet violently endeavoring, held it about six inches from his mouth; and then suddenly threw it violently against his teeth, and swallowed it with a convulsive choking, falling back on the pillow and slightly screaming. After a moment or two he walked across the room to let me look into his mouth. I still resisted the tendency towards calling it hydrophobia, and prescribing pediluvia and aperients, left him. The next day he was better, and not knowing at that time the remitting character of the disease, I was relieved of my fears. He swallowed with ease. Walked across the room and seemed livelier. His mother, however, said he had had a troubled night; seeing phantoms; boys looking in at the window, &c. Pulse frequent; no heat of skin; no lessening of bodily strength.

*Tuesday, Oct.* 10*th.*—Visited him with Dr. Oliver. Symptoms much more unfavorable. Pulse over 100. His skin not hot; eyes suffused and sleeping, or stupid looking. Involuntary shivering. Lying on his back with head thrown back. Phantasms more frequent and distressing. Could sit up without assistance. Attempts to swallow water accompanied with violent spasms, principally opisthotonic, indeed very decidedly so. Upon asking to see his tongue, it was protruded convulsively very far; the eyes being widely opened at the same time. Answered questions naturally; but with an expression of alarm. Picking his bedclothes; taking imaginary worms out of his mouth, and out of the water offered to him; and talking wildly when not spoken to. Pressure at nape caused shrieks and opisthotonos. Bowels had not been moved. Prescribed a very active cathartic; and a blister to the nape. Dr. J. Ware, who saw the patient this day, suggested chloroform. None was at hand, and it was 4 P. M. before I could visit him again, I found that at two o'clock he had commenced vomiting, and had vomited some three or four times. His countenance expressed great prostration. Extremities cold. Pulse not to be counted. Respiration not hurried. Spasms more frequent, and more easily excited. Much mucous râle. Two dejections involuntarily and unconsciously. Mind always wandering, though recalled for a moment. Attempted to administer chloroform. Much resistance to it. More tractable when his father administered it. At one time I thought him under the influence of it; but his expression was that of a dying child, and I directed it to be discontinued. The most urgent symptom now was debility. Coldness of nose and extremities. Pulse too rapid to count. Sweat on face. Sinking of features. Directed brandy and chloroform every half hour. Administering this caused spasms, but not as much horror of the fluid. On one occasion he remarked he had spilt more than half, and asked for some more. When not aroused he talked much at intervals, and then would lie quiet until a spasm came on. These were increasing and commencing with a general shiver, and then affecting the muscles of the back and extensors. They were not very violent; never raising the shoulder from the bed; but consisted simply in stretching the head, neck and the arm out; and stiffening of the trunk. About half past five I had to leave, and asked Dr. Buckminster Brown the favor to watch my patient as long as convenient. He saw him very soon after; found him lying with his eyes wide open; pupils dilated; surface heated, in a profuse sweat. Pulse feeble, fluctuating, 232 in a minute; and just afterwards 208. Every ten minutes the child was seized with a convulsive gasp, and then with a general convulsion; the head being briskly thrown or drawn to the left side and opisthotonos strongly marked. These were immediately produced, or greatly aggravated by the introduction of liquid into the mouth. He appeared to be partially sensible of what was said; would gaze on a spoon offered to him, fixing his eyes glaringly upon it as it approached his mouth, and then taking a small swallow with a convulsive gasp and struggle, was thrown into a spasm, and an expression of great suffering came over his countenance. Symptoms of depressed vitality were marked. Delirium. Dr. B. remained with him one hour and a half, say till 7. At eight I was again with him, and found the same appearances; but with greater prostration of strength, and evident symptoms of approaching dissolution. Fanning him produced convulsions. At half past 9 Dr. Brown again saw him, and reports the convulsions not so violent; legs cold to knees. Brandy and water given; but at once rejected from the mouth with frothy matter. A light produced no change on the pupils, which were widely dilated. The face was bedewed with sweat at times. Left him at 11 with the symptoms of debility fast increasing. From this time until death, the spasms were slight but easily excited, and vomiting accompanied them. He died at 2 A. M., Oct. 11th.

*Post-mortem examination with Dr. C. E. Ware.*—All organs of abdomen and chest healthy. Pericardium had a little, say ½oz. of fluid (clear) in it. Right auricle enormously distended with blood. Spinal column opened. Much venous engorgement; marrow removed from commencement of cauda equina to medulla oblongata; but nothing abnormal observed.—*From Records of Bost. Soc. for Med. Improvement. Amer. Jour. of Med. Sciences.*

